# Novel BRAF N581S mutation in mantle cell lymphoma

**DOI:** 10.1002/jha2.847

**Published:** 2024-01-17

**Authors:** Nisha Hariharan, Davsheen Bedi, Michael Y. Choi, Huan‐You Wang, Benjamin M. Heyman

**Affiliations:** ^1^ Division of Hematology/Oncology Department of Medicine University of California San Diego La Jolla California USA; ^2^ Department of Pathology University of California San Diego La Jolla California USA; ^3^ Division of Regenerative Medicine Department of Medicine University of California San Diego La Jolla California USA

**Keywords:** BRAF mutation, mantle cell lymphoma, novel

## Abstract

BRAF mutations are associated with a small number of hematologic malignancies, including hairy cell leukemia and histiocytic disorders. In addition, BRAF mutations have also been detected in low frequency in other B‐cell lymphomas, such as chronic lymphocytic leukemia and diffuse large B‐cell lymphoma, but never in mantle cell lymphoma (MCL). We present a case of a 69‐year‐old female with classic MCL harboring a BRAF^N581S^ mutation. To our knowledge, this is the first reported case of any BRAF mutation in MCL.

## 1 INTRODUCTION

BRAF is a serine/threonine kinase and a component of the mitogen‐activated protein kinase (MAPK) pathway [1]. Mutations in BRAF lead to an oncogenic form of the protein causing constitutive activation of the MAPK pathway and dysregulated cell proliferation [2]. *BRAF* ^V600E^ is the most common mutational variant and is frequently associated with solid tumors, such as melanoma, thyroid, and colorectal cancers [2]. In the realm of hematologic malignancies, *BRAF* ^V600E^ is strongly implicated as a driver mutation in the development of hairy cell leukemia (HCL) and systemic histiocytic disorders such as Langerhans cell histiocytosis (LCH) and Erdheim–Chester disease (ECD) [3, 4].

However, while BRAF mutations have been described in other hematologic malignancies, including diffuse large B‐cell lymphoma (DLBCL), they are rare outside of HCL and histiocytic disorders [4]. Additionally, the clinical significance of mutational variants outside of V600E is also unclear. We herein present a case of a 69‐year‐old female with classic MCL, found to have a *BRAF* ^N581S^ mutation. To the best of our knowledge, this is the first case of a BRAF mutation being reported in a patient with MCL.

## 2 CASE

**FIGURE 1:**
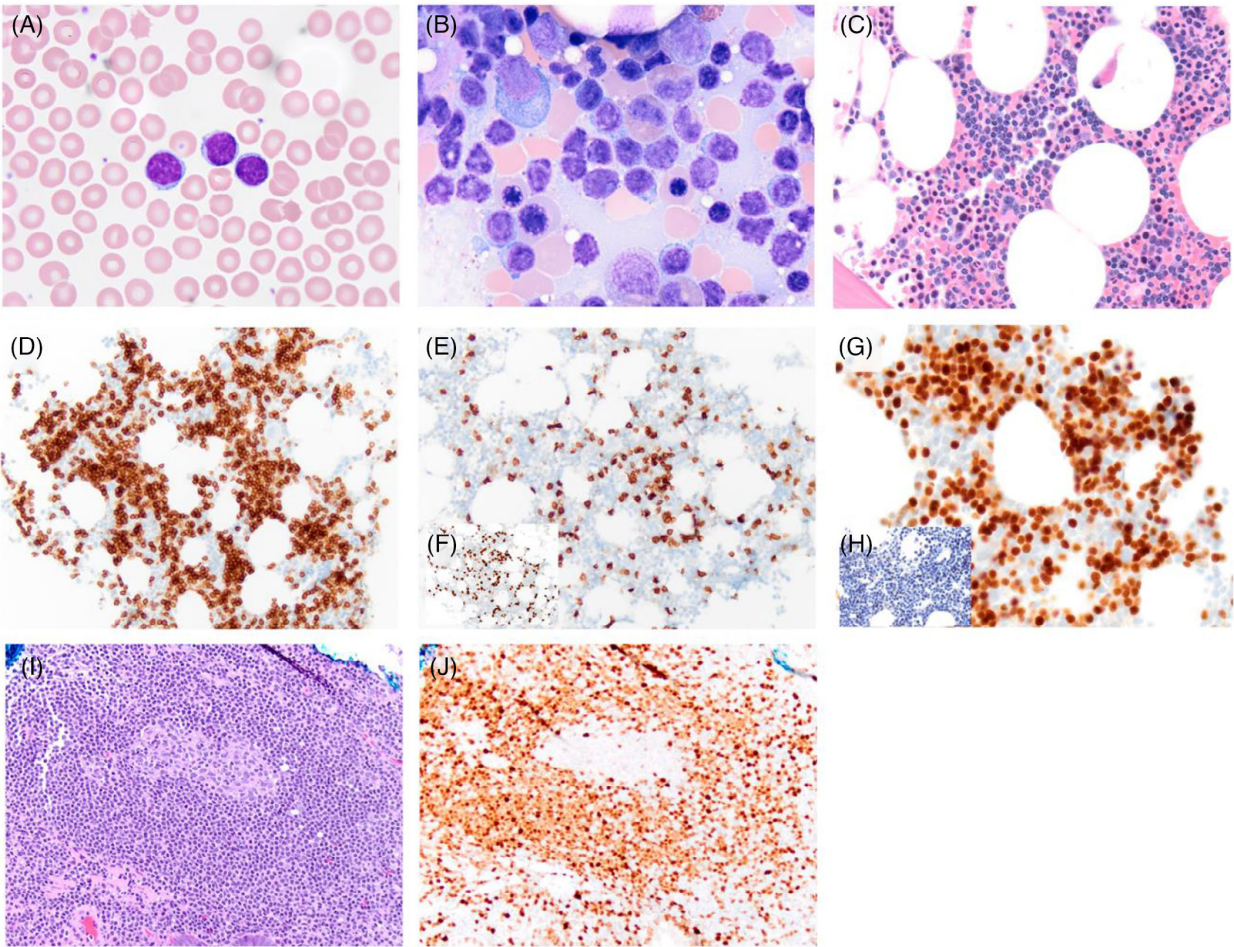
Composite microphotographs of a mantle cell lymphoma involving bone marrow (A–H) and colon (I and J). Frequent atypical small mature lymphoid cells are present in the peripheral blood (A, Wright‐Giemsa, original magnification 1000×) and bone marrow aspirate (B, Wright‐Giemsa, 1000×). The atypical lymphoid cells exhibit interstitial infiltrate of bone marrow and account for ∼50% of total cellularity (C, hematoxylin and eosin [H&E], 400×) and are positive for CD79a (D, 200×). In comparison to CD3 (E, 200×), the atypical B cells are negative for CD5 (F, inlet, 200×), but positive for BCL1 (G, 400×) albeit negative for SOX11 (H, inlet, 400×). The atypical lymphoid cells show mantle zone growth around a reactive secondary germinal center (I, 200×) and in the lamina propria of the colon and are positive for BCL1 (J, 200×).

We describe the case of a 69‐year‐old female with a history of colon polyps, pancreatic cyst, osteopenia, and MCL. She was referred to Hematology after being found to have asymptomatic leukocytosis, with an elevated white blood cell count of 25,200/mm^3^ (normal range: 4000–11,000/mm^3^) and an absolute lymphocyte count of 20,900/mm^3^ (normal range: 800–31,000/mm^3^) (Figure 1A). Her hemoglobin and platelet count were within the normal range. Peripheral blood flow cytometry revealed lambda‐restricted monotypic B cells that were negative for CD5, CD10, and CD23.

The patient subsequently underwent a bone marrow (BM) biopsy. The BM aspirate showed increased small to medium‐sized atypical lymphoid cells (Figure 1B). Flow cytometry of the BM showed 48% CD5(–)/CD10(–) kappa‐restricted monotypic B cells. Hematoxylin and eosin of the BM core biopsy demonstrated increased interstitial infiltrate of small to medium‐sized atypical lymphoid cells (Figure 1C) that were positive for CD79a (Figure 1D), but were negative for CD5 (Figure 1E) in comparison to CD3 (inlet, Figure 1F). While these cells were positive for BCL1 (Figure 1G), they were negative for SOX11 (inlet, Figure 1H). Conventional karyotyping demonstrated the presence of two clones both with t(11;14)(q13;q32), though only one clone had ring chromosome 17, leading to loss of 17p including TP53. Fluorescent in situ hybridization confirmed that t(11;14)(q13;q32) was indeed a BCL1–IGH fusion, confirming the diagnosis of CD5‐/SOX11‐classic MCL. Polymerase chain reaction of BM aspirate revealed immunoglobulin heavy chain variable region (IGHV) somatic hypermutation. Next‐generation sequencing (NGS) of the BM aspirate revealed two BCL1 mutations: E36K and Y44H with variant allele frequency (VAF) of 16% and 13%, respectively. Interestingly, NGS also revealed a *BRAF* ^N581S^ mutation with a VAF of 14%. The coding sequence showed a mutation in c.1742A>G with resulting protein p.N581S. Subsequent Immunohistochemistry (IHC) stains for BRAF were negative. Other clinically significant variants detected by NGS included a *DNMT3A* ^Y735C^ mutation, with a VAF of 4%. Computed tomography imaging at the time of diagnosis showed conspicuous, but not pathologically enlarged, bilateral level II to IV cervical lymph nodes and paraesophageal lymph nodes, with no lymphadenopathy elsewhere nor hepatosplenomegaly. A subsequent surveillance colonoscopy 19 months after the initial diagnosis demonstrated ileocecal valval involvement by MCL (Figure 1I,J).

Because the patient was asymptomatic and without cytopenias, lymphadenopathy, and splenomegaly, the decision was made to place her on watch and wait surveillance. She continues to be monitored 32 months after her initial diagnosis without indications for treatment initiation.

## 3 DISCUSSION

We describe a unique case of classic MCL with the following features: (1) the first known case of BRAF mutation in MCL, and the first known BRAF^N581S^ mutation in non‐Hodgkin lymphoma (NHL); (2) an indolent clinical course without treatment for 32 months; (3) somatic hypermutation of IGHV; and (4) negativity for both CD5 and SOX11.

BRAF mutations are rare in hematologic malignancies as compared to solid tumors, although there are two hematologic malignancies in which BRAF mutations are strikingly prominent. *BRAF* ^V600E^ mutations are found in virtually all cases of HCL (>97%) and are very common in systemic histiocytic disorders [3]. Studies estimate a V600E mutation frequency of 57%–64% in LCH patients and 54% of ECD patients [4]. BRAF mutation is now recognized as the key causal genetic event in pathogenesis of HCL, and is also thought to play a key role in pathogenesis of systemic histiocytoses [4].

However, the role of BRAF mutations in other hematologic malignancies is unclear. One study sequencing 48 HCL patients and 195 patients with other B‐cell lymphoproliferative disorders found a 100% rate of *BRAF* ^V600E^ mutations in HCL patients, and no BRAF mutations in the “other” types of B‐cell lymphoproliferative diseases, which included MCL, splenic marginal zone lymphoma, chronic lymphocytic leukemia (CLL), follicular lymphoma, DLBCL, and Burkitt lymphoma [5]. BRAF mutations have been described at low frequency in other lymphoid neoplasms. For example, one study noted 16% of 25 patients with nodal marginal zone lymphoma harboring a BRAF mutation, and just 2.8% in 138 CLL patients [6, 7].

However, unlike HCL and the systemic histiocytoses where the high frequency of BRAF mutations suggests that BRAF serves as an oncogenic driver, in other hematologic malignancies, it is unclear whether BRAF plays a role in disease pathogenesis, or if it is an incidentally noted passenger mutation. For example, one study examined in vitro response of BRAF‐mutated CLL cells to both sorafenib and PLX4720, an analog of vemurafenib. While cell death was induced by sorafenib in the BRAF‐mutated CLL cells, there was no significant effect from PLX4720 on the cells, suggesting that the clinical significance of BRAF mutations outside of HCL and histiocytic disorders requires further investigation [7]. Another study noted that of 642 CLL patients, 4.5% harbored a *BRAF* ^V600E^ mutation and appeared to have shorter survival, required more treatment, and were seen more commonly in patients with Richter transformation [8]. BRAF mutations were also seen more commonly in IGHV unmutated patients, which are typically associated with worse clinical outcomes [9]. Given that leukemic non‐nodal MCL patients share a similar cell of origin in memory B cells as CLL patients, this raises the question of whether BRAF mutations may portend the same poorer prognosis in MCL patients as well. Our patient had an indolent disease course despite the presence of a BRAF mutation, which may be related to her IGHV somatic hypermutation, as this has been associated with a less aggressive disease course in MCL [10].

Furthermore, while V600E mutation is the most common BRAF mutation, the N581S variant found in our patient is rare. N581S has been reported in a various tumors including melanoma, sarcoma, colorectal, ovarian, and lung cancers [11]. An N581I variant has also been described in marginal zone lymphoma [6]. This amino acid substitution is found in exon 15 in the BRAF kinase domain, but its effect on kinase activity is uncertain, though there is some suggestion in case reports that the N581S mutation may portend decreased sensitivity to BRAF inhibition [12].

*BRAF* ^V600E^ inhibition has made significant progress for patients with certain solid tumors, but has not been extensively studied as a treatment for hematologic malignancies. In HCL, BRAF inhibition for patients with relapsed and refractory disease can yield a high overall response rate (ORR) of 96%–100% [13]. In systemic histiocytoses, first‐line treatment with vemurafenib had an ORR of 61.5% [14]. BRAF inhibition has not been studied extensively in other lymphoid malignancies. MCL is an aggressive lymphoma with variable prognosis. For young, fit patients, standard treatment consists of cytarabine‐based chemoimmunotherapy, followed by autologous stem cell transplant and rituximab maintenance. This can lead to long remissions, but is not curative. For elderly or unfit patients, front‐line chemoimmunotherapy with bendamustine–rituximab is standard, but has shorter remissions. In the relapsed setting, Bruton tyrosine kinase inhibitors are standard but lead to responses lasting between only 1 and 2 years [15].

The reported case here is notable for several reasons. First, this is the first described case of any BRAF mutation in MCL, and second, the first described case of BRAF N581S mutation in NHL in the English literature. To the best of our knowledge, NHLs have never been treated with BRAF inhibitors. If additional reports of BRAF mutations in MCL emerge in the literature, then BRAF inhibition could represent a potential new treatment target.

## AUTHOR CONTRIBUTIONS

Nisha Hariharan and Benjamin M. Heyman conceived the idea for work and wrote the manuscript. Davsheen Bedi, Huan‐You Wang, and Michael Y. Choi analyzed the data and assisted with manuscript writing.

## CONFLICT OF INTEREST STATEMENT

The authors declare they have no conflicts of interest. Benjamin M. Heyman received research funding and/or has served as an advisor for Astrazeneca, Beigene, Oncternal Therapeutics, and Epizyme. Michael Y. Choi has received research funding and/or has served as an advisor for Abbvie, Astrazeneca, Geron, Janssen, Protagonist, Beigene, Oncternal, and Prelude.

## FUNDING INFORMATION

This study was not supported by any sponsor or funder.

## ETHICS STATEMENT

Ethical approval was not required by the UCSD institutional review board.

## PATIENT CONSENT STATEMENT

Written informed consent was obtained by the subject for publication of the details of their medical case and any accompanying images.

## CLINICAL TRIAL REGISTRATION

The authors have confirmed clinical trial registration is not needed for this submission.

## Data Availability

All data regarding this report can be found in the manuscript itself.

## References

[1] Ahmadzadeh A , Shahrabi S , Jaseb K , Norozi F , Shahjahani M , Vosoughi T , et al. BRAF mutation in hairy cell leukemia. Oncol Rev. 2014;8(2):253. 25992240 10.4081/oncol.2014.253 PMC4419648

[2] Davies H , Bignell GR , Cox C , Stephens P , Edkins S , Clegg S , et al. Mutations of the BRAF gene in human cancer. Nature. 2002;417(6892):949–954. 12068308 10.1038/nature00766

[3] Falini B , Martelli MP , Tiacci E . BRAF V600E mutation in hairy cell leukemia: from bench to bedside. Blood. 2016;128(15):1918–1927. 27554081 10.1182/blood-2016-07-418434

[4] Berres ML , Lim KP , Peters T , Price J , Takizawa H , Salmon H , et al. BRAF‐V600E expression in precursor versus differentiated dendritic cells defines clinically distinct LCH risk groups. J Exp Med. 2014;211(4):669–683. 24638167 10.1084/jem.20130977 PMC3978272

[5] Ping N , Wang Q , Wang Q , Dong S , Wu L , Xue Y , et al. Absence of BRAF V600E mutation in hematologic malignancies excluding hairy‐cell leukemia. Leuk Lymphoma. 2012;53(12):2498–2499. 22639828 10.3109/10428194.2012.695777

[6] Pillonel V , Juskevicius D , Ng CKY , Bodmer A , Zettl A , Jucker D , et al. High‐throughput sequencing of nodal marginal zone lymphomas identifies recurrent BRAF mutations. Leukemia. 2018;32(11):2412–2426. 29556019 10.1038/s41375-018-0082-4 PMC6224405

[7] Jebaraj BM , Kienle D , Buhler A , Winkler D , Dohner H , Stilgenbauer S , et al. BRAF mutations in chronic lymphocytic leukemia. Leuk Lymphoma. 2013;54(6):1177–1182. 23088640 10.3109/10428194.2012.742525

[8] Gimenez N , Martinez‐Trillos A , Montraveta A , Lopez‐Guerra M , Rosich L , Nadeu F , et al. Mutations in the RAS–BRAF–MAPK–ERK pathway define a specific subgroup of patients with adverse clinical features and provide new therapeutic options in chronic lymphocytic leukemia. Haematologica. 2019;104(3):576–586. 30262568 10.3324/haematol.2018.196931 PMC6395334

[9] Leeksma AC , Taylor J , Wu B , Gardner JR , He J , Nahas M , et al. Clonal diversity predicts adverse outcome in chronic lymphocytic leukemia. Leukemia. 2019;33(2):390–402. 30038380 10.1038/s41375-018-0215-9 PMC6718955

[10] Navarro A , Clot G , Royo C , Jares P , Hadzidimitriou A , Agathangelidis A , et al. Molecular subsets of mantle cell lymphoma defined by the IGHV mutational status and SOX11 expression have distinct biologic and clinical features. Cancer Res. 2012;72(20):5307–5316. 22915760 10.1158/0008-5472.CAN-12-1615 PMC3763938

[11] Yao TW , Zhang J , Prados M , Weiss WA , James CD , Nicolaides T . Acquired resistance to BRAF inhibition in BRAFV600E mutant gliomas. Oncotarget. 2017;8(1):583–595. 27611946 10.18632/oncotarget.11882 PMC5352180

[12] Liu Y , Zeng H , Wang K , Li Y , Tian P , Li W . Acquired BRAF N581S mutation mediated resistance to gefitinib and responded to dabrafenib plus trametinib. Lung Cancer. 2020;146:355–357. 32553555 10.1016/j.lungcan.2020.06.004

[13] Tiacci E , Park JH , De Carolis L , Chung SS , Broccoli A , Scott S , et al. Targeting mutant BRAF in relapsed or refractory hairy‐cell leukemia. N Engl J Med. 2015;373(18):1733–1747. 26352686 10.1056/NEJMoa1506583 PMC4811324

[14] Diamond EL , Subbiah V , Lockhart AC , Blay JY , Puzanov I , Chau I , et al. Vemurafenib for BRAF V600‐mutant Erdheim–Chester disease and Langerhans cell histiocytosis: analysis of data from the histology‐independent, phase 2, open‐label VE‐BASKET study. JAMA Oncol. 2018;4(3):384–388. 29188284 10.1001/jamaoncol.2017.5029 PMC5844839

[15] Wang ML , Blum KA , Martin P , Goy A , Auer R , Kahl BS , et al. Long‐term follow‐up of MCL patients treated with single‐agent ibrutinib: updated safety and efficacy results. Blood. 2015;126(6):739–745. 26059948 10.1182/blood-2015-03-635326 PMC4528064

